# Parameter inference from hitting times for perturbed Brownian motion

**DOI:** 10.1007/s10985-014-9307-7

**Published:** 2014-09-04

**Authors:** Massimiliano Tamborrino, Susanne Ditlevsen, Peter Lansky

**Affiliations:** 1 Institute of Physiology, Academy of Sciences of the Czech Republic, Videnska 1083, 142 20 Prague 4, Czech Republic; 2 Department of Mathematical Sciences, Copenhagen University, Universitetsparken 5, 2100 Copenhagen, Denmark

**Keywords:** First passage times, Maximum likelihood estimation, Wiener process, Degradation process, Effect of intervention, Survival analysis

## Abstract

A latent internal process describes the state of some system, e.g. the social tension in a political conflict, the strength of an industrial component or the health status of a person. When this process reaches a predefined threshold, the process terminates and an observable event occurs, e.g. the political conflict finishes, the industrial component breaks down or the person dies. Imagine an intervention, e.g., a political decision, maintenance of a component or a medical treatment, is initiated to the process before the event occurs. How can we evaluate whether the intervention had an effect? To answer this question we describe the effect of the intervention through parameter changes of the law governing the internal process. Then, the time interval between the start of the process and the final event is divided into two subintervals: the time from the start to the instant of intervention, denoted by $S$, and the time between the intervention and the threshold crossing, denoted by $R$. The first question studied here is: What is the joint distribution of $(S,R)$? The theoretical expressions are provided and serve as a basis to answer the main question: Can we estimate the parameters of the model from observations of $S$ and $R$ and compare them statistically? Maximum likelihood estimators are calculated and applied on simulated data under the assumption that the process before and after the intervention is described by the same type of model, i.e. a Brownian motion, but with different parameters. Also covariates and handling of censored observations are incorporated into the statistical model, and the method is illustrated on lung cancer data.

**Electronic supplementary material:**

The online version of this article (doi:10.1007/s10985-014-9307-7) contains supplementary material, which is available to authorized users.

## Introduction

Statistical inference for univariate stochastic processes from observations of hitting times, i.e. epochs when the process attains a boundary for the first time, is a common problem, see Lee and Whitmore (2006) and references therein. Here we investigate its specific variant for perturbed stochastic processes and discuss it in a general setting, presenting some of the fields in which this methodology can be applied. At a known time instant, either controlled by an experimentalist or induced by an independent external condition, an intervention is applied and the time to a given event following the intervention is measured. Assume that the intervention causes a change in the parameters of the underlying process. This scenario can be found in many fields, such as reliability theory, social sciences, finance, biology or medicine. The time course of the intervention can be interpreted as a time-varying explanatory factor in a threshold regression. Also constant and time-varying covariates can be incorporated into the underlying parametric model for the stochastic process, in the spirit of Lee et al. (2008, 2010).

A degradation process in a medical context is commonly modeled as an intrinsic, but not observable, diffusion stochastic process. With this interpretation, our model takes into account an abrupt change of medication or life style before an observable event takes place. For example, in Commenges and Hejblum (2013) the event is myocardial infarction or coronary heart disease and the degradation is the atheromatous process, which is modeled as a Brownian motion with drift, where the drift is a function of explanatory variables. Lee et al. (2008) use a time scale transformation to accommodate treatment switching in clinical trails: the total survival time from randomization is a linear combination of two event times, randomization-to-switch and switch-to-death. Here we keep the original times, but instead model the switching by a change in the drifts, which introduces a dependence structure between the two times. The interpretation in our model is that the underlying Wiener process is a model of a deterioration process, and the intervention either accelerates or slows down the risk process. Lee et al. (2010) propose a Markov Threshold regression model for time-varying covariates. The model decomposes the complete longitudinal process of a subject into a series of shorter processes based on times at which observed covariates change in value. Between two consecutive measurements, the latent process describing the health status of a subject is then approximated by a function of the observed covariates. In this paper we do not assume access to the time-course of the covariates, and the latent process is estimated only through the observed times before and after the intervention.

Similarly to the survival context in medicine, for analysing reliability of technical systems it is important to investigate damage processes. A common model is the Wiener process (Whitmore 1995; Whitmore and Schenkelberg 1997; Whitmore et al. 1998, 2012; Kahle and Lehmann 1998). In Pieper et al. (1997), changing drifts of Wiener processes describes various stress levels for a damage process. Doksum and Hoyland (1992) use a Gaussian process and inverse Gaussian distribution (IGD) to discuss a lifetime model under a step-stress accelerated life test. Nelson (2008) discusses practical issues when conducting an accelerated life test. Yu (2003) proposed a systematic approach to the classification problem where the products’ degradation paths satisfy Wiener processes. Our model fits into the above framework as follows. The degradation of a component is modeled by a Wiener process with failure corresponding to the first crossing of a certain level. The time for maintenance is independently of the time since last repair and the maintenance changes the parameters of the Wiener process. Then from measurements of the time from last repair to the time of maintenance and from the maintenance to the degradation, we deduce the effect of the maintenance on the system.

Lancaster (1972) makes effective use of the IGD in describing data on duration of strikes in UK between 1965 and 1972. The approach is via the first passage time (FPT) of an underlying Wiener process, which follows an IGD, and has also been used by Harrison and Stewart (1993) ,Desmond and Yang (2011). Again, the model studied in this paper can fit this scenario. Imagine that during a strike an important offer towards strikers is proposed. Then the time after may move on a different scale.

In neuroscience, the interval between two consecutive action potentials is often studied being related to information transfer in neurons. The Wiener process is sometimes chosen to model the subthreshold membrane potential evolution of the neuron (Gerstein and Mandelbrot 1964) and parameter estimation has been investigated (Lansky and Ditlevsen 2008). In many experiments, a stimulation (the intervention) such as a sound or a visual image is presented and the changes in electrical activity of the neuron is measured. Estimation from observations of the last action potential before the intervention and the next following it, also in presence of delayed response to the stimulus, has been investigated (Tamborrino et al. 2012, 2013). The current model also fits this framework.

The aim of this paper is to solve two problems. The first is the investigation of the joint distribution of the subintervals up to the instant of intervention, and between the intervention and the first crossing after it. This is needed for the second problem, namely the estimation of the parameters of the process before and after the intervention and testing their equality. This allows to statistically judge the effect of an intervention, if it is as intended or expected and to quantify the size, by comparing latent processes before and after intervention within subjects. The proposed modeling framework can then serve as an alternative to standard survival models, where placebo groups in a medical context have to be included in a randomized experiment to evaluate the effect of treatment. Obviously, in our model, the time to treatment and time to failure are dependent and the statistical inference is complicated by not observing the position of the process at the time of intervention. Further complications arise in the presence of censoring or truncation. Right censoring occurs if the event does not happen before the end of study, which for example is often occurring in medical studies as in the example above where a patient does not die before the end of study or is lost to follow-up. Also left censoring has to be accounted for if time of diagnosis or disease onset is unknown. Another type of missing data can occur if the event happens before the intervention, e.g. a strike ends without any political intervention or a patient dies before the beginning of a treatment. With a slightly abuse of notation we will call this truncation. These schemes can easily be incorporated into the likelihood, as long as data are available. This can be a problem under truncation: If the study is started at time of intervention, then the study population is defined as those subjects who receive the intervention, and data from before are collected retrospectively. Then it is not well-defined how many study subjects have an event before the intervention. This can bias the estimates of parameters governing the process before intervention, as will be illustrated on a data set on lung cancer. This will typically be a problem in medical studies, but not in the strike example, where for example ”strikes in UK between 1965 and 1972” is well-defined. In the neuroscience example, neither censoring nor truncation will be relevant, because the observation period typically will include many spikes both before and after the intervention, and thus, the interval containing the intervention is always fully observed.

The main contributions of the paper are the solutions to these questions in the case of a perturbed Brownian motion. A detailed guideline on how to carry out both simulation of the data and parameter estimation in the computing environment **R** (R Development Core Team 2011) is presented (see Appendices 2 and 3). Using the derived theoretical expressions, estimation could be carried out for more complicated diffusion processes.

In Sect. 2 the type of experimental data together with a description of the involved quantities and variables are presented. In Sect. 3 we describe the model, mathematically define the quantities of interest and derive the probability densities for a general diffusion process. The Brownian motion model under different assumptions on its parameters is treated in Sect. 4. The estimation procedure, accommodating for covariates and for right and left censored and truncated data, is described in Sect. 5. The performance of the maximum likelihood estimators and testing the difference between parameters are illustrated in Sect. 6 on simulated data, and finally the Veteran’s Administration lung cancer data set taken from Kalbfleisch and Prentice (1980) is analyzed in Sect. 7 and compared to previous analysis.

## Data

**Fig. 1:**
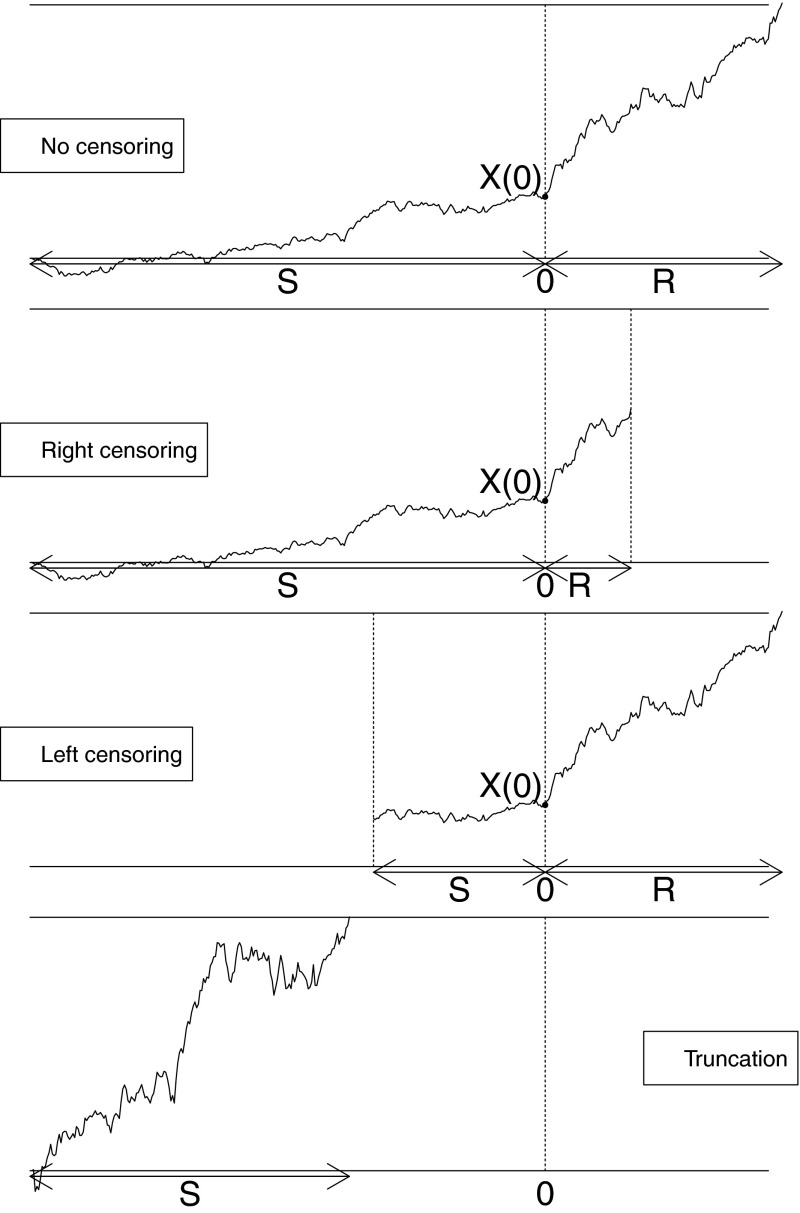
Schematic illustration of the single trial. At time 0, an intervention is applied, dividing the observed interval into two subintervals: the time $S$ upto the instant of intervention, and the time $R$ between the intervention and the first crossing after it. The random position of the process at time 0 is denoted by $X(0)$. In the fully observed case, the variables $S$ and $R$ are uncensored (*top panel*). Under right censoring, a censoring time happens before the event (*middle top panel*). Under left censoring, the start of the process is not observed (*middle lower panel*). In presence of truncation, an event happens before the intervention occurs (*lower panel*), and $R=0$

The type of experimental data and the description of the involved quantities are illustrated in Fig. 1. At a time independent of when the process started, an intervention is applied and the time the process has run as well as the time to an event after the intervention are measured. The time of the intervention is set to 0 by convenience. The intervention divides the observed interval into two subintervals: the time from the start of the process to the instant of intervention, denoted by $S$, and the time between the intervention and an event after it, denoted by $R$. Thus, the observed interval has length $S+R$. The experiment is repeated $n$ times. This allows to obtain $n$ independent and identically distributed pairs of intervals $(S_i,R_i)$, for $i=1,\ldots , n$. Note that $S_i$ and $R_i$ are not independent. A common situation for failure time data is the need to accommodate censoring or truncation in data. Left censoring happens when the time of start of the process is not observed, and right censoring when the study ends before an event occurs. In these cases either $S$ or $R$ are only known to be larger than a given value. Truncation happens if an event occurs before the intervention. In this case $R$ is undefined.

## Model and its properties

We describe the dynamics of the system by a diffusion process $X(t)$, starting at some initial value $x_0$. An event occurs when $X$ exceeds a threshold $B>x_0$ for the first time, which for now is assumed not to happen before time 0. Later this assumption will be relaxed (truncation is allowed for). The (unobserved) position of the process at time of the intervention is $X(0)$. Thus, $t$ is running in the interval $[-S,R]$ with $S,R>0$, and we assume $X(t)$ given as the solution to a stochastic differential equation

$$\begin{aligned} \left\{ \begin{array}{l} dX(t)=\nu \left( X(t),t\right) dt + \sigma \left( X(t),t\right) dW(t),\\ X(-S)=x_0, \qquad X(R)=B,\qquad X(t)<B \text{ for } t\in [-S,R), \end{array} \right. \end{aligned}$$

where $W(t)$ is a standard (driftless) Wiener process. We consider $\nu (X(t),t)=\nu _1\left( X(t)\right)$ and $\sigma (X(t),t)=\sigma _1(X(t))$ for $t<0$, and assume that the intervention causes a change in the parameters of the underlying process to $\nu (X(t),t)=\nu _2(X(t))$, and likewise for $\sigma (X(t),t)$. If there is no intervention, the standard approach is to study the FPT of $X(t)$ through the constant boundary $B$, denoted by $T$. This is the same as the intervention having no effect. Thus, define $T=S+\inf \{ t>0: X(t)\ge B| \nu _1=\nu _2 ,\sigma _1=\sigma _2 \}$. Here $T$ is not observed, but we can still consider its distribution. In case that the FPT happens before time 0 then $T=S$.

### Probability densities of $S$, $X(0)$, $R$ and $(S,R)$

It is well known from the theory of stationary point processes that the backward recurrence time $S$ is length biased, and the density is a functional of the distribution of $T$. In particular, the probability density function (pdf) of $S$ is given by (Cox and Lewis 1966)

1 $$\begin{aligned} f_{S}(s) =\frac{\bar{F}_T(s)}{\mathbb {E}[T]}, \end{aligned}$$

where $\bar{F}_T(s)=1-\mathbb {F}_T(s)=\mathbb {P}(T>s)$ denotes the survival function, and $\mathbb {E}[T]$ is the mean of $T$. The first two moments of $S$ are given by (Cox and Lewis 1966)

2 $$\begin{aligned} \mathbb {E}[S]=\frac{\mathbb {E}[T^2]}{2\mathbb {E}[T]};\qquad \text{ Var }[S]=\frac{4\mathbb {E}[T]\mathbb {E}[T^3]-3\mathbb {E}[T^2]^2}{12 \mathbb {E}[T]^2}. \end{aligned}$$

The conditional density of $X(0)$ given that $B$ has not been crossed upto time 0 is (Aalen and Gjessing 2001)

3 $$\begin{aligned} f_{X(0)}(x|s) =\frac{\frac{\partial }{\partial x} \mathbb {P}(X(0) < x, T>s)}{\mathbb {P}(T>s)}= \frac{f^a_{X(0)}(x,s)}{\bar{F}_T(s)}, \end{aligned}$$

where $f^a_{X(0)}(x,s)$ denotes the pdf of the process at time $0$ in presence of a constant absorbing boundary and given that $X(-S)=x_0$. The unconditional density of $X(0)$ is given by

4 $$\begin{aligned} f_{X(0)}(x) =\int _0^\infty f_{X(0)}(x|s)f_S(s) ds = \frac{1}{\mathbb {E}[T]}\int _0^\infty f^a_{X(0)}(x,s)ds, \end{aligned}$$

where we used (1) and (3). The variable $R$ coincides with the FPT of $X$ through the boundary $B$, when the process starts in the random position $X(0)<B$ with conditional density $f_{R|X(0)}(r|x)$. The unconditional pdf of $R$ is given by

5 $$\begin{aligned} f_R(r)=\int _{-\infty }^B f_{R|X(0)}(r|x) f_{X(0)}(x) dx. \end{aligned}$$

The joint pdf of $(S,R)$ is

6 $$\begin{aligned} f_{(S,R)}(s,r) =\frac{1}{\mathbb {E}[T]}\int _{-\infty }^B f_{R|X(0)}(r|x) f^a_{X(0)}(x,s) dx \end{aligned}$$

since

$$\begin{aligned} F_{(S,R)}(s,r)&= \int _{0}^s \mathbb {P}(R< r| S=u)f_{S}(u)du\\&= \int _{0}^s\int _{-\infty }^B \mathbb {P}(R< r|X(0)=x, S=u)f_{X(0)}(x|u)f_{S}(u)dx du\\&= \int _{0}^s\int _{-\infty }^B \int _{0}^r f_{R|X(0)}(t|x)f_{X(0)}(x|u) f_{S}(u)dt dx du\\&= \frac{1}{\mathbb {E}[T]}\int _{0}^s\int _{-\infty }^B \int _{0}^r f_{R|X(0)}(t|x) f^a_{X(0)}(x,u)dt dx du, \end{aligned}$$

where we condition on $X(0)$, then use the Markov property, and finally insert (1) and (3).

## The Wiener process

Consider a Wiener process $X$ with $\nu _1 (X(t))=\mu _1 > 0$ and $\sigma _1(X(t),t)=\sigma _1>0$ for $t<0$ and assume that the intervention causes a change in the parameters of the underlying process to $\mu _2, \sigma _2>0$. The process is space homogeneous, meaning that increments follow the same distribution independent of where we are in state space, in contrast to mean reverting processes like the Ornstein-Uhlenbeck. The FPT distribution is completely determined by two parameters, and therefore two of the four free parameters have to be fixed for identifiability. The standard approach is to let $\mu$ vary freely, and to fix two of the three parameters $x_0$, $B$ and $\sigma$. We therefore set $x_0 = 0$ without loss of generality, and also fix $B$, which is thus giving the distance the process has to travel, and is just a scaling in arbitrary units. Since $X$ is a Wiener process with positive drift, $T$ follows an IGD, $T\sim IG( B/\mu _1, B^2/\sigma _1^2)$, mean $\mathbb {E}[T] =B/\mu _1$ and variance $\text{ Var }[T]=B\sigma _1^2/\mu _1^3$ (Chhikara and Folks 1989). The pdf of $S$ follows from (1),

7 $$\begin{aligned} f_S(s)=\frac{\mu _1}{B}\left\{ \varPhi \left( \frac{B-\mu _1s}{\sqrt{\sigma ^2_1 s}}\right) -\exp \left[ \frac{2\mu _1 B}{\sigma _1^2}\right] \varPhi \left( \frac{-B-\mu _1s}{\sqrt{\sigma _1^2 s}}\right) \right\} , \end{aligned}$$

where $\varPhi (\cdot )$ denotes the cumulative distribution function of a standard normal distribution. Inserting the first three moments of $T$ into (2), we get

8 $$\begin{aligned} \mathbb {E}[S]=\frac{B\mu _1+\sigma _1^2}{2\mu _1^2};\, \text{ Var }[S]=\frac{1}{3}\left( \frac{B\mu _1+3\sigma _1^2}{2\mu _1^2}\right) ^2 \! \! ; \, \text{ CV }(S)=\frac{B\mu _1+3\sigma _1^2}{\sqrt{3}(B\mu _1 \! +\sigma _1^2)}\!, \end{aligned}$$

where $\text{ CV }(S)$ denotes the coefficient of variation of $S$, defined as the ratio between the standard deviation and the mean. The pdf of $X(0)$ in presence of a constant absorbing boundary $B$ is (Aalen and Gjessing 2001; Cox and Miller 1965; Giraudo et al. 2011; Sacerdote and Giraudo 2013)

9 $$\begin{aligned} f^a_{X(0)}(x,s)\!=\!\frac{1}{\sqrt{2\pi \sigma ^2_1s}}\left\{ \exp \left[ -\frac{(x-\mu _1 s)^2}{2 \sigma _1^2 s}\right] \!-\!\exp \left[ \frac{2\mu _1B}{\sigma _1^2}\!-\!\frac{(x-2B-\mu _1 s)^2}{2\sigma _1^2 s}\right] \right\} , \end{aligned}$$

for $x\in (-\infty , B)$. Inserting (9) into (4), we get

10 $$\begin{aligned} f_{X(0)}(x)=\frac{1}{B}\left[ \exp \left( \frac{\mu _1(x-|x|)}{\sigma _1^2}\right) -\exp \left( \frac{2\mu _1(x-B)}{\sigma _1^2}\right) \right] . \end{aligned}$$

The mean and variance of $X(0)$ are given by

11 $$\begin{aligned} \mathbb {E}[X(0)]=\frac{B\mu _1-\sigma _1^2}{2\mu _1}, \qquad \text{ Var }[X(0)]=\frac{B^2\mu _1^2+3\sigma _1^4}{12\mu _1^2}. \end{aligned}$$

The distribution of $R$ conditioned on $X(0)=x$ is $R|X(0)\sim IG\left( (B-x)/\mu _2,(B-x)^2/\sigma _2^2\right)$. Plugging this and (10) into (5), we obtain

$$\begin{aligned} f_R(r)&= \frac{\mu _2}{B}\left[ \varPhi \left( \frac{B-\mu _2r}{\sigma _2\sqrt{r}}\right) -\varPhi \left( -\frac{\mu _2 \sqrt{r}}{\sigma _2}\right) \right] \\&+\frac{\mu _2\sigma _1^2-2\mu _1\sigma _2^2}{B\sigma _1^2}\exp \left( \frac{2\mu _1r(\mu _1\sigma _2^2-\mu _2\sigma _1^2)}{\sigma _1^4}\right) \\&\times \left[ \exp \left( \frac{2\mu _1B}{\sigma _1^2}\right) \varPhi \left( -\frac{B\sigma _1^2+2r\mu _1\sigma _2^2-\mu _2 r\sigma _1^2}{\sigma _1^2\sigma _2\sqrt{r}}\right) \right. \\&\left. -\varPhi \left( -\frac{2\mu _1r\sigma _2^2-\mu _2r\sigma _1^2}{\sigma _1^2\sigma _2\sqrt{r}}\right) \right] . \end{aligned}$$

Finally, using (9) and $f_{R|X(0)}$ in (6), we get

12 $$\begin{aligned} f_{(S,R)}(s,r)&= \frac{\mu _1}{B\sqrt{2\pi [\sigma _1^2s +\sigma _2^2 r]^3}} \exp \left\{ -\frac{(B-\mu _1s-\mu _2r)^2}{2(\sigma _1^2s + \sigma _2^2r)}\right\} \nonumber \\&\quad \times \left\{ [(B-\mu _1s)\sigma _2^2 +\mu _2\sigma _1^2s]\varPhi \left( \sqrt{r}\frac{(B-\mu _1s)\sigma _2^2+\mu _2\sigma _1^2 s}{\sigma _1\sigma _2\sqrt{s(\sigma _1^2s+\sigma _2^2r)}}\right) \right. \nonumber \\&\quad -\exp \left\{ \frac{2rB(\mu _1\sigma _2^2-\mu _2\sigma _1^2)}{\sigma _1^2 (\sigma _1^2 s+\sigma _2^2 r)}\right\} [(-B-\mu _1s)\sigma _2^2\nonumber \\&\quad \left. +\mu _2\sigma _1^2s]\varPhi \left( \frac{(-B-\mu _1s)\sigma _2^2+\mu _2\sigma _1^2 s}{\sigma _1\sigma _2\sqrt{s(\sigma _1^2s+\sigma _2^2r)}}\sqrt{r}\right) \right\} . \end{aligned}$$

**Fig. 2:**
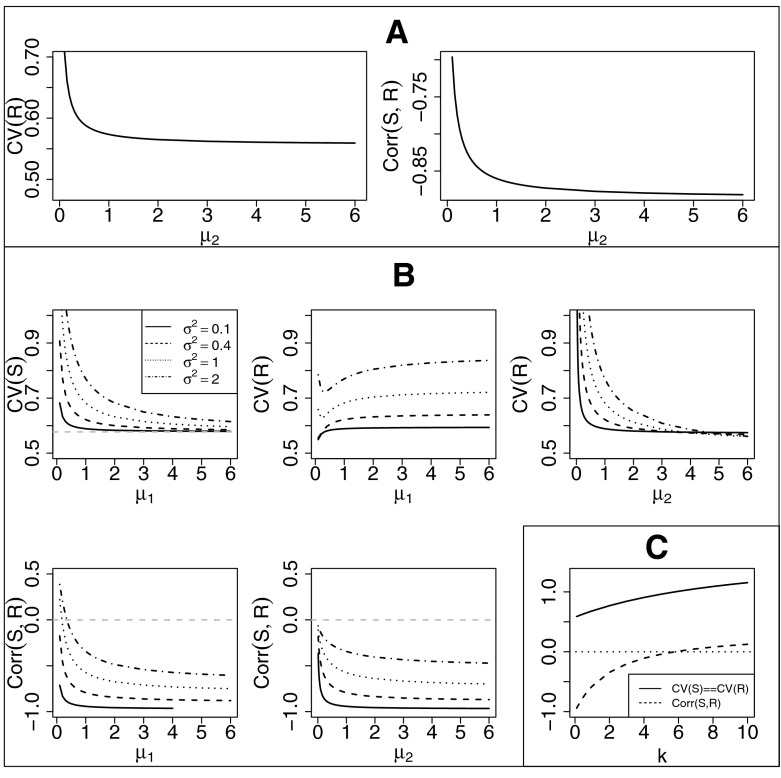
Theoretical $\text{ CVs }$ of $S$ and $R$ and $\text{ Corr }(S,R)$ as functions of $\mu _1, \mu _2$ and $k$. *Panel (A)* No further assumptions are made. The parameters are $\mu _1=1, \sigma _1^2=0.4, \sigma _2^2=0.1$, yielding an approximate $\text{ CV }(S)=0.62$. *Panel (B)* Equal variances $\sigma _1^2=\sigma _2^2=0.1, 0.4, 1$ and 2, the parameters are $\mu _1=1$ for $\mu _2\in [0.1,6]$, yielding an approximate $\text{ CV }(S)=0.59, 0.62, 0.68$ and 0.77; $\mu _2=1$ for $\mu _1\in [0.1,6]$. *Panel (C)* The variances are proportional to the drifts, i.e. $\sigma _i^2=k \mu _i, k>0$. The parameters are $\mu _1=1$ and $\mu _2=2$. Note that in this case, $\text{ CV }(S), \text{ CV }(R)$ and $\text{ Corr }(S,R)$ are the same for any value of $\mu _1$ and $\mu _2$, since they do not depend on $\mu _1$ and $\mu _2$ (see Sect. 4.1)

No closed expressions for $\text{ CV }(R)$, covariance and correlation of $S$ and $R$ are available, except for $\sigma _i^2=k \mu _i, k>0$, as described below. In Fig. 2 we illustrate $\text{ CV }(S)$ given by (8) and numerically approximate $\text{ CV }(R), \text{ Cov }(S,R)$ and $\text{ Corr }(S,R)$ for those parameter values used in Sect. 5. Note that when $\mu _2\rightarrow \infty$, the expected time for an event after the intervention goes to zero; $\mathbb {E}[R]\rightarrow 0$. Also, $\text{ Var }[R]\rightarrow 0$, whereas $\text{ CV }(R)$ does not, as shown in Fig. 2. The figure can be helpful to understand the behaviour of the estimators for different values of the parameters.

### Special case: squared diffusion coefficients proportional to the drifts

No assumptions on the relation between changes in the drift and changes in the variance of the Wiener process have been made. However, in many applications larger values of a variable are followed by a larger variance. This is formalized, for example, by the well known psychophysical Weber’s law, claiming that the standard deviation of the signal is proportional to its strength (Laming 1986). Applying this law to the IGD by relating mean and standard deviation, given prior to Eq. (7), we obtain that $\sigma ^2$ is proportional to $\mu$. An analogous result can be derived from the diffusion approximation procedure (Lansky and Sacerdote 2001). We therefore assume the squared diffusion coefficients proportional to the drift coefficients, i.e. $\sigma ^2_i=k \mu _i$, for $k>0, i=1,2$. The above expressions simplify to

13 $$\begin{aligned} \mathbb {E}[S]&= \frac{B+k}{2\mu _1},\quad \text{ Var }[S]=\frac{(B+3k)^2}{12\mu _1^2}, \quad \text{ CV }(S)=\frac{B+3k}{\sqrt{3}(B+k)},\nonumber \\ \mathbb {E}[X(0)]&= \frac{B-k}{2}, \quad \text{ Var }[X(0)]=\frac{B^2+3k^2}{12},\nonumber \\ f_R(r)&= \frac{\mu _2}{B}\left\{ \varPhi \left( \frac{B-\mu _2r}{\sqrt{k\mu _2 r}}\right) -\exp \left( \frac{2 B}{k}\right) \varPhi \left( \frac{-B-\mu _2r}{\sqrt{k \mu _2 r}}\right) \right\} =\frac{\bar{F}_{T^*}(r)}{\mathbb {E}[T^*]},\nonumber \\ \end{aligned}$$

where $T^*$ denotes the FPT through $B$ of the Wiener process starting in 0 with drift $\mu _2$ and diffusion coefficient $\sqrt{k\mu _2}$. Note that $R$ is distributed as the forward recurrence time of $T^*$, as well as $S$ is distributed as the backward recurrence time of $T$. Thus

14 $$\begin{aligned} \mathbb {E}[R]=\frac{B+k}{2\mu _2},\qquad \text{ Var }[R]=\frac{(B+3k)^2}{12\mu _2^2},\qquad \text{ CV }(R)=\frac{B+3k}{\sqrt{3}(B+k)}. \end{aligned}$$

Interestingly, $\text{ CV }(S)=\text{ CV }(R)$ and they only depend on $k$ and not on the specific values of the coefficients. The joint pdf of $S$ and $R$ is

15 $$\begin{aligned} f_{(S,R)}(s,r)&= \frac{\mu _1\mu _2}{\sqrt{2\pi k (\mu _1s+\mu _2r)^3}}\exp \left( -\frac{(B-\mu _1s-\mu _2r)^2}{2k(\mu _1s+\mu _2r)}\right) \nonumber \\&= \frac{\mu _1\mu _2}{B}f_{IG(B,B^2/k)}(\mu _1s+\mu _2r), \end{aligned}$$

and the covariance and correlation of $S$ and $R$ are

16 $$\begin{aligned} \text{ Cov }(S,R)&= \mathbb {E}[SR]-\mathbb {E}[S]\mathbb {E}[R]=\frac{3k^2-B^2}{12\mu _1\mu _2},\end{aligned}$$

17 $$\begin{aligned} \text{ Corr }(S,R)&= \frac{\text{ Cov }(S,R)}{\sqrt{\text{ Var }[S]\text{ Var }[R]}}=\frac{3k^2-B^2}{(B+3k)^2}, \end{aligned}$$

see Appendix 1 for detailed derivation. Note that the correlation can be positive, null or negative, depending on whether $0<k<B/\sqrt{3}, k=B/\sqrt{3}$ or $k>B/\sqrt{3}$, respectively. Moreover, $\text{ Corr }(S,R)\rightarrow -1$ as $k\rightarrow 0$, i.e. $\sigma _i^2\rightarrow 0$, while $\text{ CV }(S)=\text{ CV }(R)\rightarrow \sqrt{3}$ and $\text{ Corr }(S,R)\rightarrow 1/3$ as $k\rightarrow \infty$, i.e. $\sigma _i^2\rightarrow \infty , i=1,2$.

## Parameter estimation

The aim of this paper is the estimation of the parameters of $X$ from a sample of $n$ independent observations of $(S,R)$, and testing if the intervention has an effect by the hypothesis $H_0: \mu _1=\mu _2$. To take into account possible censoring and truncation, denote the censoring variables $C_i^r$, the right censoring time for subject $i$, and $C_i^l$, the left censoring time defined as the maximum time that can be observed before the intervention for subject $i$. If truncation happens, then $T=S$ and $R$ is undefined and arbitrarily set to 0. We consider data of the form $\{(s_i,r_i, \delta _i^l,\delta _i^r,\nu _i)\}_{i=1}^n$. Here $\delta _i^l$, $\delta _i^r$ and $\nu _i$ are indicator variables for left and right censoring and truncation, respectively:

$$\begin{aligned} \delta _i^l&= \left\{ \begin{array}{cl} 0 &{} \text{ if } \text{ there } \text{ is } \text{ left } \text{ censoring }\\ 1&{} \text{ if } \text{ there } \text{ is } \text{ not } \text{ left } \text{ censoring } \end{array} \right. , \quad \delta _i^r=\left\{ \begin{array}{cl} 0 &{} \text{ if } \text{ there } \text{ is } \text{ right } \text{ censoring }\\ 1&{} \text{ if } \text{ there } \text{ is } \text{ not } \text{ right } \text{ censoring } \end{array} \right. ,\\ \nu _i&= \left\{ \begin{array}{cl} 0 &{} \text{ if } \text{ there } \text{ is } \text{ truncation, } \text{ i.e. } T=S\\ 1&{} \text{ if } \text{ there } \text{ is } \text{ not } \text{ truncation, } \text{ i.e. } T>S \end{array} \right. . \end{aligned}$$

Here $s_i$ is the observation of $\min (S_i,C_i^l)$ if $T_i> S_i$, it is the observation of $S_i$ if $T_i= S_i$ and $C_i^l\ge S_i$ (truncation), and it is the time passed from entrance in the study to time of event if $T_i= S_i$ and $C_i^l<S_i$ (truncation and left-censoring). Finally $r_i$ is the observation of $\min (R_i,C_i^r)$. Note that if $\nu _i = 0$ then $R$ plays no role and we set $\delta _i^r=1$. We will always assume independent censoring, defined as the risk of the event being independent of the censoring times. The $(s_i, r_i,\delta _i^l,\delta _i^r,\nu _i)$’s, $i=1,\ldots , n$ are independent and identically distributed, and for independent censoring, the log-likelihood is (Kalbfleisch and Prentice 1980)

18 $$\begin{aligned} l_{(S,R)}(\phi )&= \sum _{i=1}^n \delta _i^l\delta _i^r \nu _i \log f_{(S,R)}(s_i,r_i) + \sum _{i=1}^n(1-\delta _i^l) \delta _i^r\nu _i \log \int _{0}^{s_i} f_{(S,R)}(s,r_i)ds \nonumber \\&\quad + \sum _{i=1}^n(1-\delta _i^r) \delta _i^l\nu _i \log \int _{r_i}^\infty f_{(S,R)}(s_i,r)dr + \sum _{i=1}^n(1-\nu _i) \delta _i^l \log f_T(s_i) \nonumber \\&\quad + \sum _{i=1}^n(1-\nu _i)(1-\delta _i^l) \log \int _{0}^{s_i} f_T(s)ds \nonumber \\&\quad + \sum _{i=1}^n(1-\delta _i^r) (1-\delta _i^l)\nu _i \log \int _{r_i}^\infty \int _{0}^{s_i} f_{(S,R)}(s,r)dsdr. \end{aligned}$$

The first term on the right hand side of (18) evaluates the contributions for full observations without neither censoring nor truncation, the second and third terms are the contributions for left and right censored observations, the fourth term corresponds to truncation, the fifth term to both truncation and left censoring, and the last term corresponds to both left and right censoring.

The model can easily be extended to incorporate baseline covariates $z_1, \ldots , z_p$. If the effects are linear in the drifts it takes the following form for a subject $i$:

$$\begin{aligned} \mu _{1i}= z_{1i} \beta _1 + \cdots + z_{pi} \beta _p, \end{aligned}$$

where $\beta _j, j=1, \ldots , p$, are regression parameters to estimate. The intervention will cause a change given by $m$ further covariates, e.g. $m$ different types of treatment. Then

$$\begin{aligned} \mu _{2i}= z_{1i} \beta _1 + \cdots + z_{pi} \beta _p + z_{p+1,i} \beta _{p+1}+ \cdots + z_{p+m,i} \beta _{p+m}=Z_i \beta . \end{aligned}$$

The parameters enter implicitly in the log-likelihood (18) through the dependence on $\mu _1$ and $\mu _2$. In the simplest case where $\mu _1$ and $\mu _2$ are the same for all subjects we have $p=m=1$, and $\beta = (\beta _1,\beta _2)^T$ determines the drifts.

The maximum likelihood estimator $\hat{\phi }=(\hat{\beta },\hat{\sigma }^2_1, \hat{\sigma }^2_2)$ is found by numerically maximizing (18) (see Appendix 3 for detailed description). An approximate 95 % confidence interval (CI) for $\phi _i$ is given by $\hat{\phi }_i \pm 1.96\ \text{ SE }(\hat{\phi }_i)$, where $\text{ SE }$ is the asymptotic standard error given by $\text{ SE }(\hat{\phi }_i)=\sqrt{I_{ii}(\hat{\phi })^{-1}/n}$, where $I(\phi )$ is the Fisher information matrix (Cramer 1946), which we numerically approximate (see Appendix 3). To test the hypothesis $H_0:\mu _1=\mu _2$ we perform a likelihood ratio test at a 5 % significance level, evaluating it in a chi-squared distribution with $m$ degrees of freedom. The test statistic is $-2\log [ L_0(\hat{\phi }_0)/L_\mathrm{full}(\hat{\phi })]$, where $L_0$ and $L_\mathrm{full}$ denote the likelihood functions of the null and full (alternative) model evaluated in the estimated parameters $\hat{\phi }_0=(\hat{\mu },\hat{\sigma }_1^2,\hat{\sigma }^2_2)$ and $\hat{\phi }=(\hat{\mu }_1,\hat{\mu }_2,\hat{\sigma }_1^2,\hat{\sigma }^2_2)$ under the hypotheses $\mu =\mu _1=\mu _2$ (corresponding to $\beta _{p+1}= \cdots = \beta _{p+m}=0$) and $\mu _1\ne \mu _2$, respectively.

In the following the performance of the estimators is checked on simulated data in a simple set-up both without and with right censoring, and then on a data set with a more complicated structure, incorporating covariate effects. This is the Veteran’s Administration lung cancer data set taken from Kalbfleisch and Prentice (1980), which is analyzed and results are compared.

## Monte Carlo simulation study

Here we briefly summarize the main results from the simulation study. An extended treatment and further figures can be found in the online material accompanying the paper. In the simulations we are mainly concerned with illustrating the performance of the estimators. It is of interest to evaluate the effect of the variability and correlation of $S$ and $R$ on estimation, to evaluate sample sizes needed for the asymptotic results of tests and CIs to be valid, to illustrate different special submodels which simplify estimation, and finally to evaluate how much information is gained on parameters of $S$ by taking into account observations of $R$.

In the simulations, three scenarios are considered: no information about the parameters is available, i.e. all parameters can vary freely; we assume equal variances $\sigma _1^2=\sigma _2^2=\sigma ^2$; or we assume $\sigma ^2_i=k\mu _i$, as in Sect. 4.1. That is, we want to estimate either $\phi =(\mu _1,\sigma _1^2,\mu _2,\sigma _2^2), \phi =(\mu _1,\mu _2,\sigma ^2)$ or $\phi =(\mu _1,\mu _2,k)$. We assume both the parametric form of the underlying process and the relations between parameters, if any, to be known. It can be discussed if these assumptions are realistic. Equality of diffusion coefficients, or the assumption of variance proportional to the mean, can be checked by likelihood ratio test.

**Table 1:** Averages, empirical and asymptotic SEs and CPs in percentage over 1,000 estimates of $\phi =(\mu _1,\sigma _1^2,\mu _2,\sigma _2^2)$ for $n=100$, when $\mu _1=1, \sigma _1^2=0.4, \mu _2=0.1$, and $\sigma _2^2 = 0.026, 0.059, 0.094$, or 0.131, yielding an approximate $\text{ CV }(R)=0.60, 0.65, 0.70$ or 0.75, respectively

	Average	Empirical	Asymptotic		Average	Empirical	Asymptotic	
CV(R)	$\text{ of } \hat{\mu }_1$	$\text{ SE }(\hat{\mu }_1)$	$\text{ SE }(\hat{\mu }_1)$	$\text{ CP }(\hat{\mu }_1)$	$\text{ of } \hat{\sigma }_1^2$	$\text{ SE }(\hat{\sigma }^2_1)$	$\text{ SE }(\hat{\sigma }^2_1)$	$\text{ CP }(\hat{\sigma }^2_1)$
0.60	0.9998	0.0405	0.0397	94.7	0.39962	0.1079	0.1027	91.6
0.65	1.0020	0.0438	0.0428	93.7	0.4016	0.1213	0.1154	91.3
0.70	1.0023	0.0468	0.0441	94.5	0.3983	0.1315	0.1198	91.8
0.75	1.0020	0.0458	0.0449	94.9	0.3989	0.1388	0.1251	91.4

	Average	Empirical	Asymptotic		Average	Empirical	Asymptotic	
CV(R)	$\text{ of } \hat{\mu }_2$	$\text{ SE }(\hat{\mu }_2)$	$\text{ SE }(\hat{\mu }_2)$	$\text{ CP }(\hat{\mu }_2)$	$\text{ of } \hat{\sigma }_2^2$	$\text{ SE }(\hat{\sigma }^2_2)$	$\text{ SE }(\hat{\sigma }^2_2)$	$\text{ CP }(\hat{\sigma }^2_2)$
0.60	0.1003	0.0032	0.0032	94.8	0.0256	0.0083	0.0080	92.7
0.65	0.1001	0.0044	0.0043	93.7	0.0578	0.0154	0.0145	91.9
0.70	0.1000	0.0053	0.0051	93.7	0.0926	0.0221	0.0212	92.1
0.75	0.1001	0.0058	0.0058	95.5	0.1290	0.0288	0.0278	92.9

In all cases, $\text{ CV }(S)=0.62$

*Parameters vary freely* Details about the settings of parameters, sample sizes and number of repetitions can be found in the online material, and are also given in Table 1, where averages and empirical SEs of the estimates, as well as medians of the asymptotic SEs and the coverage probabilities of the CIs are reported. All estimators appear unbiased and with acceptable SEs. Not surprisingly, the performance improves when the CV of $R$ decreases. This holds also for $\hat{\mu }_1$ and $\hat{\sigma }_1^2$, highlighting the dependence between $S$ and $R$: a large variability after the intervention deteriorates estimation of parameters governing the process before the intervention. Coverage probabilities of drift parameters are close to the desired 95 %, whereas the diffusion parameters $\sigma _1^2$ and $\sigma _2^2$ need a larger $n$.

**Fig. 3:**
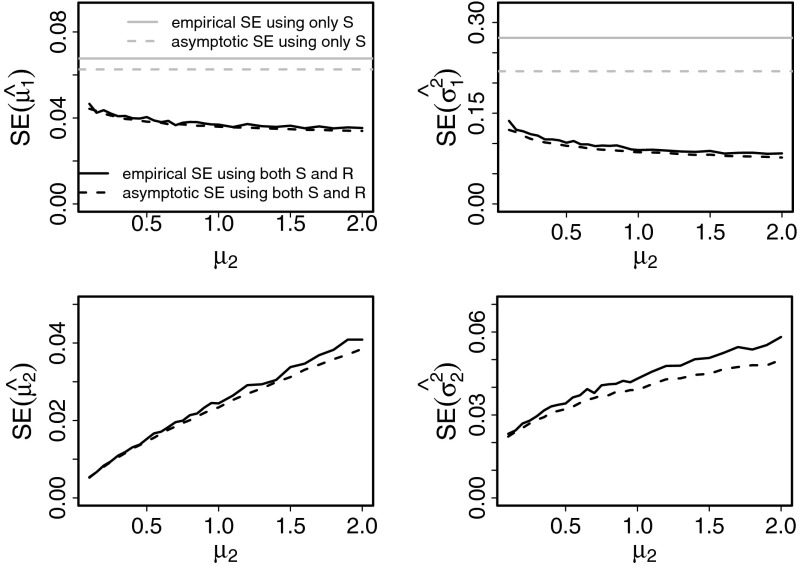
Empirical and asymptotic SEs over 1,000 estimates of $(\mu _1,\sigma _1^2,\mu _2,\sigma _2^2)$ for $n=100$ as a function of $\mu _2$ when no assumptions on the parameters are made. The parameters are $\mu _1=1, \sigma _1^2=0.4, \sigma _2^2=0.1$, yielding an approximated $\text{ CV }(S)=0.62$. *Full lines* empirical SEs. *Dashed lines* asymptotic SEs. *Colors* correspond to the SEs of the estimators obtained by either maximizing $l_{(S,R)}$ (*black lines*), or maximizing $\log f_S$ (*gray lines*), respectively

A relevant question is how much, if at all, the estimators of $\mu _1$ and $\sigma _1^2$ improve by considering the more complicated likelihood based on Eq. (12) compared to the simple likelihood based on Eq. (7), where information from $R$ is ignored. The estimates of $\mu _1$ and $\sigma _1^2$ obtained from observations of $(S,R)$ outperform those obtained only from observations of $S$, as can be seen comparing both their empirical and asymptotic SEs in Fig. 3. When $\mu _2$ increases, the performance of $\hat{\mu }_1$ and $\hat{\sigma }^2_1$ improve and that of $\hat{\mu }_2$ and $\hat{\sigma }_2^2$ get worse even if $\text{ CV }$ of $R$ decrease. Moreover, the difference between the empirical and the asymptotic SEs for $\hat{\mu }_2$ and $\hat{\sigma }_2^2$ increases with $\mu _2$, and thus, for large $\mu _2$, a larger sample size is needed for asymptotics to be valid. Otherwise the empirical and asymptotic SEs are approximately equal, and thus the asymptotic values appear acceptable for inference purposes.

*Equal variances* When $\sigma _1^2 = \sigma _2^2=\sigma ^2$, the behavior of the estimators is similar, and with equal variances we can more easily analyze the behavior of the drift estimators as functions of the parameters. All estimators improve when $\sigma ^2$ decreases, since that reduces the variability of both $S$ and $R$. The performance of $\hat{\mu }_i$ improves while that of $\hat{\mu }_j$ gets worse when $\mu _j$ increases, for $i,j=1,2$ and $i\ne j$. Interestingly, the performance of $\hat{\sigma }^2$ seems to be constant with respect to $\mu$, unless $\sigma ^2$ is large. A likelihood ratio test for testing the hypothesis $H_0:\mu _1=\mu _2$ performs well for Type I error when $n=100$ for different sizes of $\sigma ^2$. Not surprisingly, the power of the test decreases when $\sigma ^2$ increases.

*Variance proportional to the mean* Assume $\sigma _i^2 = k \mu _i$, for $k>0$. As expected from the theoretical results in Sect. 4.1, the performance of $\hat{\mu }_1$ and $\hat{\mu }_2$ appears similar, and it does not depend on $\mu _2$ and $\mu _1$, respectively. Interestingly, the asymptotic SE of $\hat{k}$ depends neither on $\mu _1$ nor on $\mu _2$, but only on $k$. This may be due to the fact that neither the $\text{ CVs }$ of $S$ and $R$ nor their correlation depend on $\mu _1$ and $\mu _2$, see Eqs. (13), (14) and (17).

*Right truncation* The effect of censoring on the estimation of $\phi$ is illustrated in the online material, where boxplots of the estimates are reported for different percentage of right censored data and sample sizes. As expected, the performance of $\hat{\phi }$ gets worse when the percentage of right censored data increases and thus a larger sample size is needed.

## Veterans’ Administration lung cancer data

The model is applied on the *Veterans’ Administration lung cancer data set* from Kalbfleisch and Prentice (1980), available in the **R**-package ”survival” with the name ”veteran”. In this trial, males with advanced inoperable lung cancer were randomized to either a standard or test chemotherapy. The randomization time is the time of intervention. The primary endpoint for therapy comparison was time to death. This is a standard survival analysis data set. The following variables were recorded:

1. Disease duration: Time in months from diagnosis to randomization (observations of $S$). We transform to units of days by multiplying by 30.4.
2. Survival lifetime: Time in days from randomization to death (observations of $R$).
3. Treatment: standard, test.
4. Histological type of tumor: squamous, small, adeno, large cell.
5. A measure at randomization of the patient’s performance status (Karnofsky rating); 10–30 completely hospitalized, 40–60 partial confinement, 70–99 able to care for oneself. We call it *karno* and transform to 100-karno.
6. Age in years of the patient.
7. Prior therapy: no, yes.
8. Indicator for right censoring (observations of $\delta ^r$)

No information about death of patients before the beginning of the treatment is available, and thus it is not possible to correct for possible truncation. Only 9 of the 137 survival times were right censored, and none were left censored.

The aim of the study is to compare types of treatment and histological types of tumor. A positive component for a given covariate means a higher $\mu$ and thus increased risk. A negative component implies protection. Indeed, the best treatment and the less dangerous type of tumors should have the (expected) highest survival time and thus the lowest value of $\mu _2$, since for $X(0)=x$ is $\mathbb {E}[R|X(0)]=(B-x)/\mu _2$. Furthermore, it is of interest to compare treatment against no treatment, that is, the difference between $\mu _1$ and $\mu _2$, in particular, to judge whether any of the two treatments has an effect with respect to no treatment.

Assuming $\sigma ^2=\sigma _1^2=\sigma _2^2$, we estimate $\phi =(\beta ,\sigma ^2)$ by numerically maximizing (18), as detailed in Appendix 3. We let $\mu _1$ depend on cell type (4 categories, parametrized by absolute levels and no intercept), age (continuous variable) and whether prior therapy has been applied (dichotomous variable), thus $p=6$. Performance status at intervention time does not influence $\mu _1$, since this is measured after the time course of $S$. This is also confirmed by a likelihood ratio test for testing the hypothesis $H_0: \beta _{\mathrm{karno}\, \mathrm{in}\, \mu _1}=0$ yielding a $p$ value of 0.40. Note that performance status can be considered a proxy of risk status at time of intervention, that is, of $X(0)$. Therefore, performance status was transformed to 100-karno for more readily interpretation. By including this variable in $\mu _2$, it will (hopefully) correct for unmeasured confounders in $\mu _1$ by taking into account the actual status at time of intervention, so that the estimates of treatment effect are indeed due to treatment. Thus, performance status (continuous variable) and treatment (2 categories, both as *changes* with respect to $\mu _1$, i.e. with respect to no treatment) are added to $\mu _2$, and thus $m=3$. This implies an extra parameter compared to standard models because the time before the intervention, corresponding to no treatment, is included. In standard models this would require inclusion of an extra randomized group with placebo. Estimates and $\chi ^2$-values are reported in Table 2.

**Table 2:** Estimates of $\beta$ for all regressor variables and asymptotic $\chi ^2$ statistics

	Full model		Reduced model	
Regressor variable	$\hat{\beta }$	$\chi ^2$ value	$\hat{\beta }$	$\chi ^2$ value
Performance status (100-karno)	0.0014	23.12	0.0014	23.62
Age (years)	0.0001	0.30		
Prior therapy	$-$0.0146	17.09	$-$0.0147	17.30
Cell type				
Squamous	0.0284		0.0341	
Small	0.0379		0.0431	
Adeno	0.0522		0.0576	
Large	0.0346	17.13	0.0396	16.64
Treatment			$-$0.0231	5.37
Test	$-$0.0215			
Standard	$-$0.0277	0.44		

The variance estimate is $\hat{\sigma }^2=0.2151$ in the full model and 0.2173 in the reduced model, obtained by removing age as covariate, and merging the two treatment groups

Since treatment estimates are negative, treatment increases survival time. This information is missing in standard survival models, unless a placebo group is included in the study. A likelihood ratio test for testing $H_0: \beta _{\mathrm{standard}}=\beta _{\mathrm{test}}$ shows no statistical difference between treatment types ($p=0.51$). Age is not statistical significant either, whereas histological cell types, performance status and prior theory are statistical significant. Results for the reduced model without age and merging the two treatment groups, are reported in Table 2. These results agree with those in Kalbfleisch and Prentice (1980). In their paper, Weibull and Log-normal regression models were fitted to these data, with survival lifetime as dependent variable and disease duration prior to entry to the clinical trial, treatment (one category for the difference between test and standard treatment), cell types (large as reference level and three categories), age and prior therapy as covariates. An important difference is that they include disease duration (the variable $S$) as a covariate, whereas we include it as a driving part of the model to interpret the entire disease development. They do not find it statistical significant, whereas the test $\mu _1=\mu _2$ (i.e. $\beta _{karno}=\beta _{treatment}=0$) is strongly significant ($\chi ^2= 34.98$). This might be due to the strong significance of performance status, but also a test only of treatment effect (i.e. $\beta _{treatment}=0$) yields $\chi ^2= 5.37 \ (p=0.02)$. Furthermore, the estimate of $\mu _1$ might be strongly downward biased due to non reported deaths before the beginning of the treatment, which might also bias the regression coefficient in the analysis by Kalbfleisch and Prentice (1980). If this is the case, the treatment effect is larger than what the study shows. This is a general problem of missing data when the amount of truncation is not reported. To fully evaluate the treatment effect this information (or an estimate thereof) is needed, or a placebo randomization group should be included in the study design. An important advantage of the present model is that it allows to evaluate treatment effect as such, whereas the model of Kalbfleisch and Prentice (1980) only evaluates the difference between treatment types.

**Fig. 4:**
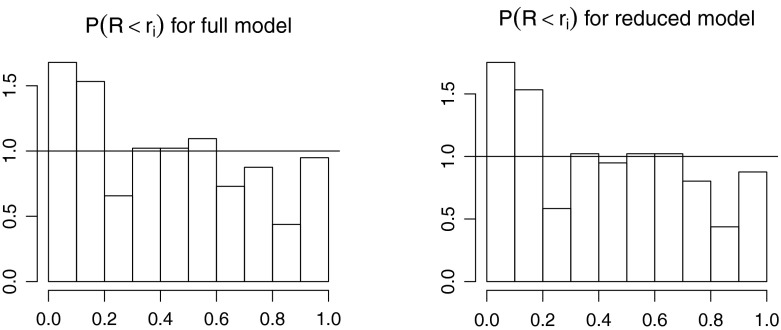
Histogram of uniform residuals from the full (*left panel*) and reduced (*right panel*) fitted models

To check the model, $\mathbb {P}(R<r_i)$ was calculated for all subjects in the fitted model. Under the model, these should be standard uniformly distributed. A histogram is shown in Fig. 4, which appear acceptable both for the full and the reduced model.

## Conclusion

In any study where an intervention is applied, the most natural question arising is whether it has an effect, and if this is the case if it is the intended effect and to quantify the size. Here, the effect is reflected in the change of the time to an observable event. However, in many studies there is no apparent information available about what such a time would have been if no intervention had been applied. In this paper we solve the problem by comparing the time to the intervention and the time to the final event. The parameters of the underlying process are identified and statistically compared to judge the presence and size of an effect. The method represents a potential tool in all the experimental or observational situations where direct measurements of the time course of the underlying process are not available, but only the qualitative changes are observable through times of observable events.

An essential assumption in our approach is that the intervention time is independent of the underlying process. This is a strong assumption and probably not fulfilled in many cases. It is difficult to avoid this assumption, unless the dependence structure is specifically modeled, which is prone to imply even stronger assumptions that might be more difficult to check or fulfil. Nevertheless, in many applications we believe it to be a reasonable assumption. In the neuroscience example when analysing neuronal spike data, the assumption is absolutely reasonable, because the time of intervention (e.g. start of stimulation) is independent of the neuronal activity, where many spikes occur both before and after the intervention. In this case neither censoring nor truncation is relevant. Also in the reliability of technical systems the assumption will often be reasonable, where an intervention is applied to the entire production at the same time, independent of how each component is evolving at that moment. However, in many medical contexts it will of course not be realistic that the intervention time is independent of disease status, and careful reservations have to be taken for possible bias in estimates. In some examples the assumption might be reasonable, though, or it might be possible to include some corrections at intervention time as done in the data example. The analysis corrects both for prior therapy as well as for performance status at intervention time. This last covariate hopefully corrects for any (or most of) the dependence as well as unmeasured confounders, where the disease state might influence the decision of whether a patient should enter the study or not and thus be randomized to one of the treatments. In this application the most serious problem is that data from before the intervention are collected retrospectively from those patients having an intervention, and thus, no information is available about possible deaths before the intervention time. We therefore expect that the estimate of the drift before the intervention is downward biased (only those surviving until intervention are kept in the analysis), and the effect of treatment might be larger than the analysis shows. In other medical examples, the assumption is fully justified. For example imagine a transplant intervention, where start is defined by being approved for a transplant, final event is death, and the intervention is the transplant. Then the intervention time will depend on when a matching organ is available, which will be independent of the disease progress in a particular patient. Here truncation (death before the transplant) will probably be present, but it can easily be corrected for if data on deaths before the intervention are available, which is also a reasonable assumption. The strike example is the most problematic, since a political decision of an intervention will likely depend on the status of the strike. In that case proper care should be taken to include possible covariates, which can hopefully correct for some of the incurred bias, such as media coverage or other social factors.

### Electronic supplementary material

Below is the link to the electronic supplementary material.

ESM 1 (PDF 120 kb)

## Appendix

### 1. Covariance and correlation of $S$ and $R$ when $\sigma _i^2=k\mu _i$

Let $P\sim IG(B,B^2/k)$, and thus $\mathbb {E}[P]=B$. Then, using (15), we have

19 $$\begin{aligned} \mathbb {E}[SR]&= \int _0^\infty \int _0^\infty sr f_{(S,R)} dr ds = \int _0^\infty \frac{s\mu _1 }{B} \int _0^\infty \mu _2 r f_{P}(\mu _1s+\mu _2r)dr ds\nonumber \\&= \int _0^\infty \frac{s\mu _1}{B}\int _{\mu _1s}^\infty \frac{1}{\mu _2} (t-\mu _1s) f_P(t) dt ds\nonumber \\&= \frac{1}{\mu _1\mu _2B}\int _0^\infty u\int _u^\infty (t-u)f_P(t)dt du\nonumber \\&= \frac{1}{\mu _1\mu _2B}\int _0^\infty u\int _u^\infty tf_P(t)dt du-\frac{1}{\mu _1\mu _2B}\int _0^\infty u^2\bar{F}_p(u) du. \end{aligned}$$

Calculating the integral in *dt* by parts, we get

20 $$\begin{aligned} \int _u^\infty t f_P(t) dt=[-t\bar{F}_P(t)]|_u^\infty +\int _u^\infty \bar{F}_p(t) dt=u\bar{F}_P(u)+\int _u^\infty \bar{F}_p(t) dt, \end{aligned}$$

where $-t\bar{F}_P(t)\rightarrow 0$ when $t\rightarrow \infty$ because $\bar{F}(t)=o(t^{-1})$ as $t\rightarrow \infty$. Define now a variable $Q$ by

$$\begin{aligned} f_Q(t)=\frac{\bar{F}_P(t)}{\mathbb {E}[P]}=\frac{\bar{F}_P(t)}{B}. \end{aligned}$$

Then, inserting (20) into (19) and simplifying the resulting expression, we obtain

21 $$\begin{aligned} \mathbb {E}[SR]&= \frac{1}{\mu _1\mu _2 B}\int _0^\infty u \int _u^\infty \bar{F}_P(t) dt du=\frac{1}{\mu _1\mu _2}\int _0^\infty u \int _u^\infty f_Q(t)dt du\nonumber \\&= \frac{1}{\mu _1\mu _2}\int _0^\infty u \bar{F}_{Q}(u)du. \end{aligned}$$

Similarly, let $Z$ be a variable defined by $f_Z(u)=\bar{F}_Q(u)/\mathbb {E}[Q]$. Then (21) becomes

22 $$\begin{aligned} \mathbb {E}[SR]=\frac{\mathbb {E}[Q]}{\mu _1\mu _2}\int _0^\infty u \frac{\bar{F}_Q(u)}{\mathbb {E}[Q]}du= \frac{\mathbb {E}[Q]}{\mu _1\mu _2} \mathbb {E}[Z], \end{aligned}$$

where

$$\begin{aligned} \mathbb {E}[Z]=\frac{1}{2}\mathbb {E}[Q]+\frac{1}{2}\frac{\text{ Var }[Q]}{\mathbb {E}[Q]}, \end{aligned}$$

see Eqs. (1) and (2). Mimicking the calculations done for $S$ in (13), we obtain $\mathbb {E}[Q]=(B+k)/2, \text{ Var }[Q]=(B+3k)^2/12$. Plugging them into $\mathbb {E}[Z]$ first and then (22), and simplifying the resulting expression, we get

$$\begin{aligned} \mathbb {E}[SR]=\frac{B^2+3Bk+3k^2}{6 \mu _1\mu _2}. \end{aligned}$$

Finally, (16) follows using (13) and (14).

### 2. Simulation in **R**

To simulate $(s_i,r_i), i=1,\ldots , n$ we proceed as follows. We simulate $s_i$ by applying the inverse transforming sampling to the cumulative distribution function of $S$, which is obtained by numerically integrating (1) using the function integrate in **R**. We obtain $s_i$ by simulating $u_i$ from a uniform distribution on $[0,1]$, and solving $F_{S}(s_i)-u_i=0$ with respect to $s_i$ by means of the function uniroot in **R**. To obtain an observation $r_i$ from $R$ we first simulate $x$, i.e. the position $X(0)$ of the process at the time of intervention. We use the inverse transforming sampling to the distribution of $X(0)$, obtained by integrating (3) with respect to $x$, i.e. $F_{X(0)}(x|s)=F^a_{X(0)}(x,s)/\mathbb {P}(T>s)$. Because $X$ is a Wiener process, $F^a_{X(0)}(x,s)$ is given by (9),

$$\begin{aligned} F^a(x,s)= \varPhi \left( \frac{x-\mu _1s}{\sqrt{\sigma ^2_1 s}}\right) -\exp \left[ \frac{2\mu _1B}{\sigma _1^2}\right] \varPhi \left( \frac{x-2B-\mu _1s}{\sqrt{\sigma ^2_1 s}}\right) . \end{aligned}$$

Using $x$, an observation $r_i$ from $R$ is drawn from $IG((B-x)/\mu _2,(B-x)^2/\sigma _2^2)$. We obtain $1\le l\le n$ right censored observations of $R$ by simulating from a uniform distribution in $(0,r_j)$, for $j=1,\ldots , l$.

### 3. Estimation of $\phi$ and $I(\phi )$ in **R**

Since the parameter values of $\mu _1,\mu _2, \sigma _1$ and $\sigma _2$ need to be positive, maximizing the log-likelihood is a constrained optimization problem. When minimizing $-l_{(s,r)}$ by means of the function optim, we penalize negative values of $\mu _1,\mu _2, \sigma _1$ and $\sigma _2$ by returning $10^{10}$.

Since $l_{(s,r)}$ is a complicated function of $\phi$, it can frequently happen that it has several local maxima. To find the global maximum, sensible starting values are paramount. The starting value $\phi _0$ for the iterations is chosen by the following strategy:

- **Monte Carlo simulation study.** Obtain $\mu _1^*, \sigma _1^{2*}$ by maximizing the log-likelihood $\log f_S$ from $s_i, i=1,\ldots , n$, with starting values given by means of moment estimation of $S$; plug $\mu _1^*, \sigma _1^{2*}$ into (11) to estimate the expected position at the time of intervention, i.e. $\hat{x}=\widehat{\mathbb {E}[X(0)]}$; using $r_i$ and $\hat{x}$, obtain $\mu _2^*, \sigma _2^{2*}$ as moment estimators for $\mu _2$ and $\sigma _2^2$ when $R|X(0)\sim IG((B-\hat{x})/\mu _2, (B-\hat{x})^2/\sigma _2^2)$, i.e.
  23 $$\begin{aligned} \mu _2^*=\frac{B-\hat{x}}{\bar{r}}, \qquad \sigma _2^{2*}=\frac{\text{ emp.var }(R) \mu _2^{3*}}{B-\hat{x}}, \end{aligned}$$
  where $\bar{r}$ denotes the average of the observations $r_i$. Alternatively, $\mu _2^*$ and $\sigma _2^*$ may be the maximum likelihood estimator (Chhikara and Folks 1989). Then $\phi _0=(\mu _1^*,\sigma _1^{2*},\mu _2^*,\sigma _2^{2*})$ is the starting value. When the variances are equal, the starting value is $\phi _0=(\mu _1^*, \sigma _1^{2*},\mu _2^*)$. When the variance is proportional to the mean, obtain $\mu _1^*, k^{*}$ by maximizing the log-likelihood $\log f_S$ from $s_i, i=1,\ldots , n$, with starting values given by means of moment estimation of $S$ through (13); obtain $\mu _2^*$ as moment estimator for $\mu _2$ from (14), i.e. $\mu _2^*=(B+k^*)/2 \bar{r}$. Then set $\phi _0=(\mu _1^*,\mu _2^*,k^*)$.
- **Veterans’ Administration lung cancer data**. We choose $\beta _\mathrm{cell}^*=0.01$ for each of the four cell types, $\beta _\mathrm{age}^*=0.0001$, $\beta _\mathrm{prior}^*=\beta _\mathrm{Performance}^*=0.01$, $\beta _\mathrm{standard}^*=\beta _\mathrm{test}^*=0.1, \sigma ^{2*}=0.1$ and set $\phi _0=(\beta ^*,\sigma ^{2*})$.

To reduce the influence of the starting value in the optimization procedure, we proceed as follows. Once that $\phi _0$ has been computed or set, we carry out the estimation procedure, and then we use the obtained estimate $\hat{\phi }$ as a new starting value $\phi _0$. We repeat this procedure until $\phi _0$ and the estimated parameters yield approximately the same value of $-\log f_{(S,R)}$.

Often an explicit expression for the inverse of the Fisher information $I(\phi )^{-1}$ is not available, but it can be numerically evaluated. In the Monte Carlo simulation study, we calculate the $d\times d$ matrix $I(\phi )/n$, for $d=4$ when no assumptions are made and $d=3$ when $\sigma _1^2=\sigma _2^2$ or $\sigma _i=k \mu _i$ using the option hessian=TRUE in the optim function. Since $I(\phi )$ is symmetric, positive definite square matrix, we invert it by means of its Cholesky decomposition. We first use the function chol to compute the Cholesky factorization and then chol2inv to invert it.
