# Supplementary material for: Parameter inference from hitting times for perturbed Brownian motion
Source: Lifetime Data Anal. 2014 Sep 4;21(3):331–52. doi: 10.1007/s10985-014-9307-7 (PMC4464758; doi:10.1007/s10985-014-9307-7)
Supplement: Supplementary file 1 — ESM 1 (PDF 120 kb) [file 10985_2014_9307_MOESM1_ESM.pdf]

## Online material for the paper
## PARAMETER INFERENCE FROM HITTING TIMES FOR PERTURBED BROWNIAN MOTION

### 1 Monte Carlo simulation study

In this online material we extend the description of the results of the simulations with further details. In the simulations we are mainly concerned with illustrating the performance of the estimators. It is of interest to evaluate the effect of the variability and correlation of $S$ and $R$ on estimation, to evaluate sample sizes needed for the asymptotic results of tests and confidence intervals to be valid, to investigate the influence of right censored data on estimation, to illustrate different special submodels which simplify estimation, and finally to evaluate how much information is gained on parameters of $S$ by taking into account observations of $R$.

In the simulations, three scenarios are considered: no information about the parameters is available, i.e. all parameters can vary freely; we assume equal variances $\sigma_1^2 = \sigma_2^2 = \sigma^2$; or we assume $\sigma_i^2 = k\mu_i$, as in Section 4.1 of the paper. That is, either $\phi = (\mu_1, \sigma_1^2, \mu_2, \sigma_2^2), \phi = (\mu_1, \mu_2, \sigma^2)$ or $\phi = (\mu_1, \mu_2, k)$ are estimated. We assume both the parametric form of the underlying process and the relations between parameters, if any, to be known. It can be discussed if these assumptions are realistic. Equality of diffusion coefficients, or the assumption of variance proportional to the mean, can be checked by likelihood ratio test. Parameter values are chosen such that the mean of $T$ in the case of no intervention is five times its standard deviation. This is obtained by setting $B = 10, \mu_1 = 1, \sigma_1^2 = 0.4$, yielding $\mathbb{E}[T] = 10$, and $\mathrm{Var}[T] = 4$. Then $\mu_1$ and $\sigma_1^2$ are varied to investigate different regimes of the model. Then the effect of the intervention is varied through the parameters $\mu_2$ and $\sigma_2^2$. Samples of size $n = 100$ are simulated, and for each set of parameter values, we repeat simulation of data set and estimation 1000 times, obtaining 1000 statistically independent trials. To illustrate the effect of right censored data, we choose the most general scenario, i.e. parameters vary freely.

| CV(R) | Average of $\hat{\mu}_1$ | Empirical SE($\hat{\mu}_1$) | Asymptotic SE($\hat{\mu}_1$) | CP($\hat{\mu}_1$) | Average of $\hat{\sigma}_1^2$ | Empirical SE($\hat{\sigma}_1^2$) | Asymptotic SE($\hat{\sigma}_1^2$) | CP($\hat{\sigma}_1^2$) |
|---|---|---|---|---|---|---|---|---|
| 0.60 | 0.9998 | 0.0405 | 0.0397 | 94.7 | 0.39962 | 0.1079 | 0.1027 | 91.6 |
| 0.65 | 1.0020 | 0.0438 | 0.0428 | 93.7 | 0.4016 | 0.1213 | 0.1154 | 91.3 |
| 0.70 | 1.0023 | 0.0468 | 0.0441 | 94.5 | 0.3983 | 0.1315 | 0.1198 | 91.8 |
| 0.75 | 1.0020 | 0.0458 | 0.0449 | 94.9 | 0.3989 | 0.1388 | 0.1251 | 91.4 |

| CV(R) | Average of $\hat{\mu}_2$ | Empirical SE($\hat{\mu}_2$) | Asymptotic SE($\hat{\mu}_2$) | CP($\hat{\mu}_2$) | Average of $\hat{\sigma}_2^2$ | Empirical SE($\hat{\sigma}_2^2$) | Asymptotic SE($\hat{\sigma}_2^2$) | CP($\hat{\sigma}_2^2$) |
|---|---|---|---|---|---|---|---|---|
| 0.60 | 0.1003 | 0.0032 | 0.0032 | 94.8 | 0.0256 | 0.0083 | 0.0080 | 92.7 |
| 0.65 | 0.1001 | 0.0044 | 0.0043 | 93.7 | 0.0578 | 0.0154 | 0.0145 | 91.9 |
| 0.70 | 0.1000 | 0.0053 | 0.0051 | 93.7 | 0.0926 | 0.0221 | 0.0212 | 92.1 |
| 0.75 | 0.1001 | 0.0058 | 0.0058 | 95.5 | 0.1290 | 0.0288 | 0.0278 | 92.9 |

**Table 1** Averages, empirical and asymptotic SEs and CPs in percentage over 1000 estimates of $\phi = (\mu_1, \sigma_1^2, \mu_2, \sigma_2^2)$ for $n = 100$, when $\mu_1 = 1, \sigma_1^2 = 0.4, \mu_2 = 0.1$, and $\sigma_2^2 = 0.026, 0.059, 0.094$, or $0.131$, yielding an approximate $\text{CV}(R) = 0.60, 0.65, 0.70$ or $0.75$, respectively. In all cases, $\text{CV}(S) = 0.62$.

Coverage probabilities (CPs) were calculated, defined as the probability that the CI covers the true value, to evaluate the performance of the CIs. The CP should be close to $1 - \alpha$, where $\alpha = 0.05$ is the significance level, and the CI should be narrow for a reliable estimator.

The computing environment **R** has been used to carry out both the simulations of $(s_i, r_i)$, including right censored data where relevant, and the parameter estimation. A description of the simulation procedure is reported in Appendix B of the paper.

*Parameters vary freely* We choose $\mu_1 = 1, \sigma_1^2 = 0.4$ and thus $\text{CV}(S) = 0.62$. First we fix $\mu_2$ and vary $\sigma_2^2$, then we fix $\sigma_2^2$ and let $\mu_2$ vary. In the first case, we fix $\mu_2 = 0.1$, implying that the intervention slows down the process, since $\mu_2 < \mu_1$. To obtain $\text{CV}(R) = 0.6, 0.65, 0.7$ or $0.75$, we set $\sigma_2^2 = 0.026, 0.059, 0.094$, or $0.131$.

Averages and empirical SEs of the estimates, as well as medians of the asymptotic SEs and the CPs of the CIs are reported in Table 1. All estimators appear unbiased and with acceptable SEs. Not surprisingly, the performance improves when the CV of $R$ decreases. This holds also for $\hat{\mu}_1$ and $\hat{\sigma}_1^2$, highlighting the dependence between $S$ and $R$: a large variability after the intervention deteriorates estimation of parameters governing the process before the intervention. Coverage probabilities of drift parameters are close to the desired 95%, whereas the diffusion parameters $\sigma_1^2$ and $\sigma_2^2$ need a larger $n$.

In the second case, we let $\mu_2$ vary in the interval $[0.1, 10]$, and fix $\sigma_2^2 = 0.1$. Here the response to the intervention either slows down or accelerates the process, depending on whether $\mu_2 < \mu_1$ or $\mu_1 < \mu_2$, respectively.

A relevant question is how much, if at all, the estimators of $\mu_1$ and $\sigma_1^2$ improve by considering the more complicated likelihood based on eq. (12) compared to the simple likelihood based on eq. (7), where information from $R$ is ignored. All estimators appear unbiased (figures not shown). The estimates of $\mu_1$ and $\sigma_1^2$ obtained from observations of $(S, R)$ outperform those obtained only from observations of $S$, as can be seen comparing both their empirical and asymptotic SEs in Fig. 1. When $\mu_2$ increases, the performance of $\hat{\mu}_1$ and

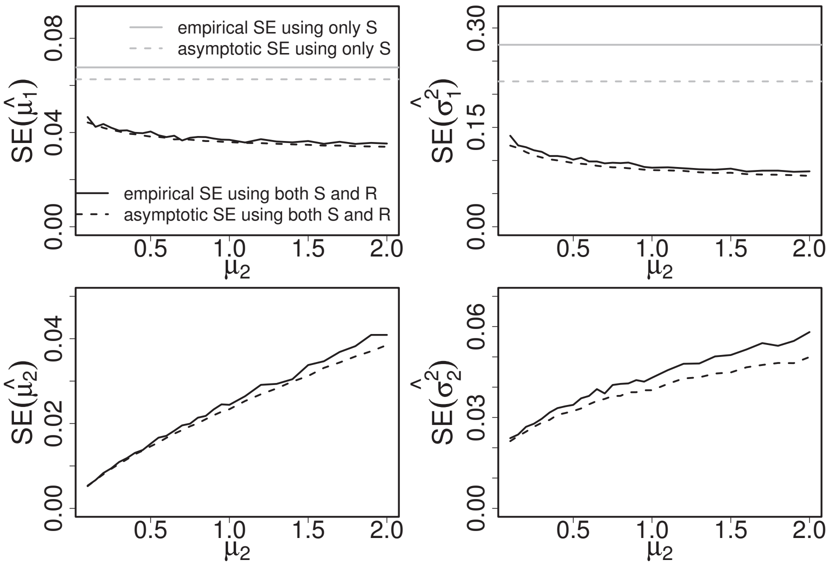


**Fig. 1** Empirical and asymptotic SEs over 1000 estimates of $(\mu_1, \sigma_1^2, \mu_2, \sigma_2^2)$ for $n = 100$ as a function of $\mu_2$ when no assumptions on the parameters are made. The parameters are $\mu_1 = 1, \sigma_1^2 = 0.4, \sigma_2^2 = 0.1$, yielding an approximated $\mathrm{CV}(S) = 0.62$. Full lines: empirical SEs. Dashed lines: asymptotic SEs. Colors correspond to the SEs of the estimators obtained by either maximizing $l_{(S,R)}$ (black lines), or maximizing $\log f_S$ (gray lines), respectively.

$\hat{\sigma}_1^2$ improve and that of $\hat{\mu}_2$ and $\hat{\sigma}_2^2$ get worse even if CV of $R$ decrease. Moreover, the difference between the empirical and the asymptotic SEs for $\hat{\mu}_2$ and $\hat{\sigma}_2^2$ increases with $\mu_2$, and thus, for large $\mu_2$, a larger sample size is needed for asymptotics to be valid. Otherwise the empirical and asymptotic SEs are approximately equal, and thus the asymptotic values appear acceptable for inference purposes. In the following we only report the asymptotic values.

*Equal variances* When $\sigma_1^2 = \sigma_2^2 = \sigma^2$, we put $\sigma^2 = 0.1, 0.4, 1$ and 2, respectively, with either $\mu_1 = 1$ and $\mu_2 \in [0.1, 10]$ or $\mu_2 = 1$ and $\mu_1 \in [0.1, 10]$. The variability of the estimators for different values of $\mu_1$ and $\mu_2$ is reported in Fig. 2, where the SEs of the estimators are plotted against $\mu_2$. The estimators appear unbiased (results not shown). All of them improve when $\sigma^2$ decreases, since that reduces the variability of both $S$ and $R$. The performance of $\hat{\mu}_i$ improves while that of $\hat{\mu}_j$ gets worse when $\mu_j$ increases, for $i, j = 1, 2$ and $i \neq j$. Interestingly, the performance of $\hat{\sigma}^2$ seems to be constant with respect to $\mu$, unless $\sigma^2$ is large.

A likelihood ratio test is performed for testing the hypothesis $H_0 : \mu_1 = \mu_2$ at a 5% significance level and the percentage of rejections of $H_0$ as a function of $\mu_2$ is reported in Fig. 3. If $\mu_1 = \mu_2$, the percentage should be around 5%, while if $\mu_1 \neq \mu_2$, the percentage represents the power of the test, i.e. the probability of correctly rejecting the null hypothesis, and it should be as

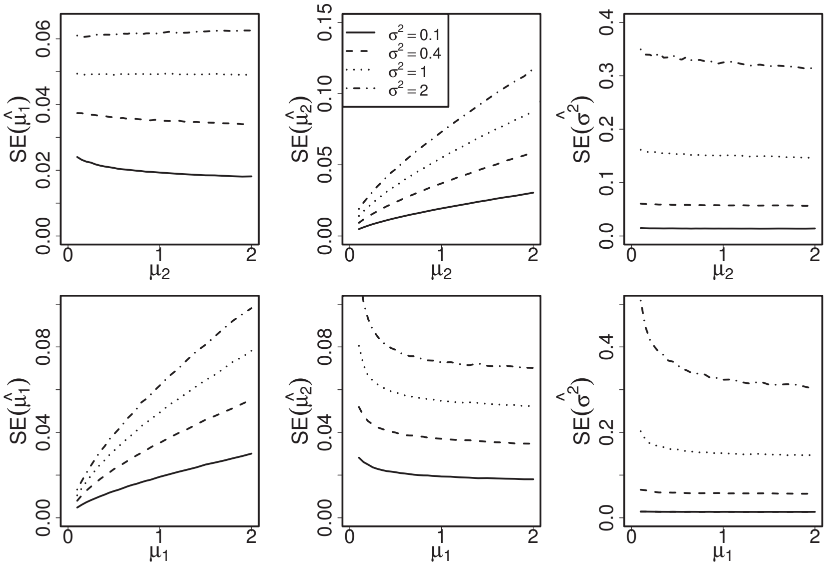


**Fig. 2** Asymptotic SEs over 1000 estimates of $(\mu_1, \mu_2, \sigma^2)$ for $n = 100$ as a function of $\mu_2$ (upper panels) and of $\mu_1$ (lower panels) for equal variances, $\sigma_1^2 = \sigma_2^2 = \sigma^2$. In both cases, $\sigma^2 = 0.1$ (full lines), 0.4 (dashed lines), 1 (dotted lines) and 2 (dotted-dashed lines). In the upper panel, $\mu_1 = 1$ (upper panels) yielding an approximate $\mathrm{CV}(S) = 0.59, 0.62, 0.68$ and $0.77$, respectively, and in the lower panel $\mu_2 = 1$.

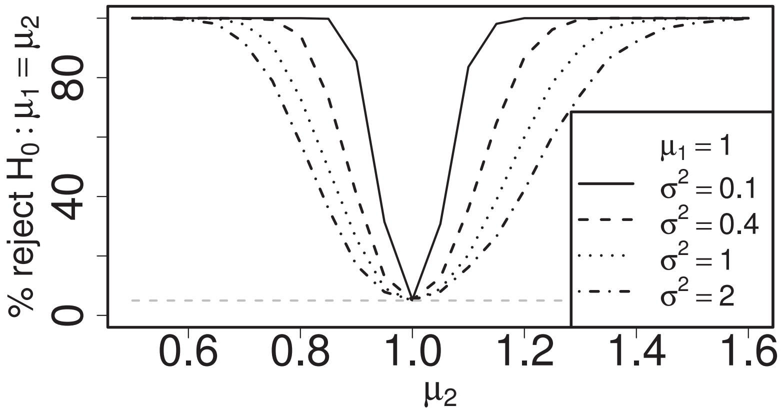


**Fig. 3** Percentage of rejections, using the likelihood ratio test at 5% significance level of the null hypothesis $H_0 : \mu_1 = \mu_2$ as a function of $\mu_2$ for equal variances, $\sigma^2 = \sigma_1^2 = \sigma_2^2$. The parameters are $\mu_1 = 1, \sigma^2 = 0.1$ (full line), 0.4 (dashed line), 1 (dotted line) and 2 (dashed-dotted line).

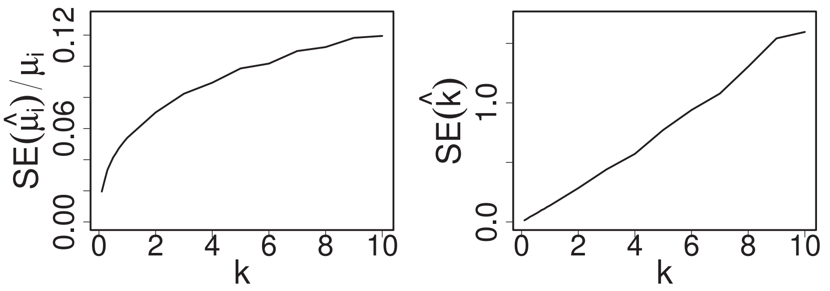


**Fig. 4** Asymptotic SEs over 1000 estimates of $\mu_i$ and $k$ for $n = 100$ rescaled by $\mu_i$ as a function of $k$ when the variance is proportional to the mean, $\sigma_i^2 = k\mu_i, i = 1, 2$. The parameters are $\mu_1 = 1$ and $\mu_2 = 2$. The results for SE$(\hat{\mu}_1)/\mu_1$ and SE$(\hat{\mu}_2)/\mu_2$ are almost indistinguishable. The same results hold for other combinations of $(\mu_1, \mu_2)$ and are therefore not reported.

high as possible. When $\mu_1 = \mu_2 = 1$, this percentage is around 5% for all values of $\sigma^2$, suggesting that $n = 100$ is sufficient for asymptotics to be valid. Not surprisingly, the power of the test decreases when $\sigma^2$ increases, but it is worthwhile noting that it is larger than 50% when $|\mu_1 - \mu_2| > 0.2$ and around 100% if $|\mu_1 - \mu_2| \geq 0.4$, indicating a satisfactory performance of the test.

*Variance proportional to the mean* Assume $\sigma_i^2 = k\mu_i$, for $k > 0$. The parameter values are $k \in [0.1, 10]$ and $\mu_1, \mu_2 \in \{0.1, 1, 2\}$. The performance of the estimators is reported in Fig. 4, where SE$(\hat{\mu}_i)/\mu_i$ and SE$(\hat{k})$ are plotted against $k$. Also in this case, estimators appear unbiased (results not shown). As expected from the theoretical results in Section 4.1, the performance of $\hat{\mu}_1$ and $\hat{\mu}_2$ appears similar, and it does not depend on $\mu_2$ and $\mu_1$, respectively. Interestingly, the asymptotic SE of $\hat{k}$ depends neither on $\mu_1$ nor on $\mu_2$, but only on $k$. This may be due to the fact that neither the CVs of $S$ and $R$ nor their correlation depend on $\mu_1$ and $\mu_2$, see eqs. (13), (14) and (17).

*Right censoring* To illustrate the effect of censoring, we consider the more general scenario, i.e. no assumptions on the parameters, and $l = 5, 25, 50\%$ right censored data for different sample sizes, namely $n = 100, 200, 500$ and 1000. The parameters are $\mu_1 = 1, \sigma_1^2 = 0.4, \mu_2 = 2$ and $\sigma_2^2 = 0.1$ chosen as worst case scenario giving the worst performance in terms of SEs of $\hat{\mu}_2$ and $\hat{\sigma}_2^2$ for non-censored data, as shown in Fig. 1. Boxplots of $\hat{\phi}$ for different values of $l$ and $n$ are reported in Fig. 5. The boxes contain the estimates between the 1st and the 3rd quartiles, while the 2nd quartile, i.e. the median of the estimates, is marked with the black horizontal line. The bars show the range of the estimates, except the outliers, defined to be the points outside the bars that are more than 1.5 times the interquartile range from the box. As expected, the performance of $\hat{\phi}$ gets worse when the percentage of right censored data increases and thus a larger sample size is needed.

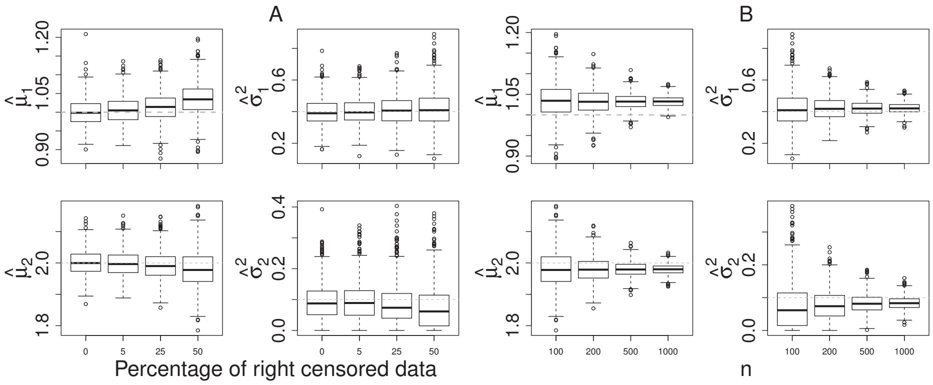


**Fig. 5** Boxplots over 1000 estimates of $(\mu_1, \sigma_1^2, \mu_2, \sigma_2^2)$ as a function of percentage of right censored data and $n$ when no assumptions on the parameters are made. The parameters are $\mu_1 = 1, \mu_2 = 2, \sigma_1^2 = 0.4, \sigma_2^2 = 0.1$. Left Figure A: $n = 100$ and different percentage of right censored data. Right Figure B: 25% of right censored data and different values of $n$.
